# Methylxanthines: Potential Therapeutic Agents for Glioblastoma

**DOI:** 10.3390/ph12030130

**Published:** 2019-09-07

**Authors:** Daniel Pérez-Pérez, Iannel Reyes-Vidal, Elda Georgina Chávez-Cortez, Julio Sotelo, Roxana Magaña-Maldonado

**Affiliations:** 1 PECEM, Faculty of Medicine, National Autonomous University of México, México City 04510, Mexico; 2 Neuroimmunology and Neuro-oncology Unit, National Institute of Neurology and Neurosurgery, México City 14269, Mexico

**Keywords:** brain tumors, natural alkaloids, drug repositioning

## Abstract

Glioblastoma (GBM) is the most common and aggressive primary brain tumor. Currently, treatment is ineffective and the median overall survival is 20.9 months. The poor prognosis of GBM is a consequence of several altered signaling pathways that favor the proliferation and survival of neoplastic cells. One of these pathways is the deregulation of phosphodiesterases (PDEs). These enzymes participate in the development of GBM and may have value as therapeutic targets to treat GBM. Methylxanthines (MXTs) such as caffeine, theophylline, and theobromine are PDE inhibitors and constitute a promising therapeutic anti-cancer agent against GBM. MTXs also regulate various cell processes such as proliferation, migration, cell death, and differentiation; these processes are related to cancer progression, making MXTs potential therapeutic agents in GBM.

## 1. Introduction

Glioblastoma (GBM) is the most aggressive and most frequent primary malignant tumor of the central nervous system (CNS) [1]. It occurs more frequently in men and in people older than 55 years [2]. Pathological characteristics of GBM include cellular heterogeneity, angiogenesis, high proliferation rate, and increased migratory capacity [3]. The treatment of GBM consists of surgical resection, radiotherapy, chemotherapy, and novel therapies such as alternating electrical fields [4]. Nevertheless, even with these novel therapeutic approaches, the overall survival of patients is around 20.9 months [5].

Activation of aberrant signaling pathways in GBM may promote the survival of neoplastic cells [6] and can provide new therapeutic targets. For example, phosphodiesterase (PDE) dysregulation in GBM leads to the survival, proliferation, and dedifferentiation of neoplastic cells [7]. Targeting this pathway could therefore be a valuable tool therapy for GBM.

Due to the ineffectiveness of the current GBM therapy, diverse natural compounds have been evaluated as chemotherapeutic agents, for example, resveratrol, curcumin, epigallocatechin 3-gallate gallate (EGCC), and others. These agents can be used as adjuvant therapy to improve standard treatments [8]. Curcumin is effective alone and combined with standard therapy in glioma cells; it also induces neural differentiation of human pluripotent embryonal carcinoma cells [9]. The EGCC in green tea induces cell death and reduces cellular proliferation and invasion in diverse glioma cell lines. It also enhances the efficacy of chemotherapy and radiotherapy in GBM [10]. Among these agents are the methylxanthines (MXTs); these are natural compounds with anti-cancer properties, the induction of apoptosis, the reduction of cellular migration, the arrest of the cell cycle, and the inhibition of PDEs. All these properties constitute therapeutic targets against GBM. Information about the effectiveness and mechanisms of the action of MTXs on GBM is summarized in the present review.

**Figure 1:**
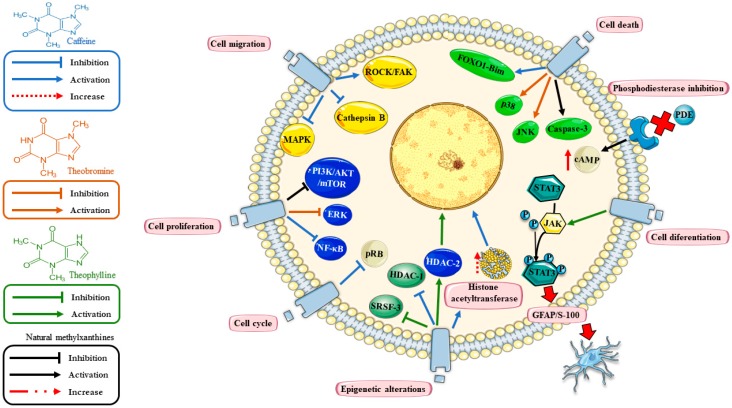
Mechanisms of action of methylxanthine (MTXs) in glioblastoma (GBM). The figure shows common pathways regulated by MTXs (black arrows) including inhibition of phosphodiesterases (PDEs) (reducing the levels of cyclic Adenosine Monophosphate (cAMP)); regulation of cell proliferation (PI3K/AKT-mTOR); and cell death (caspase 3). Particularly, caffeine (blue arrows) promotes cell death (FOXO1-Bim); cell migration (by these proteins ROCK/FAK pathway, MAPK and cathepsin B); cell proliferation (NF-kB); cell cycle (pRB) and epigenetic mechanisms (HAT‒histone acetyltransferases and HDAC-1). Theobromine (orange arrow) regulates cell proliferation (ERK) and apoptosis (JNK and p38). Theophylline (green arrow) regulates cell differentiation (JAK/STAT3 pathway), the epigenetic mechanism (HDAC-2) and alternative splicing (SRSF3). The figure was designed using Servier Medical Art ©.

There are several natural or synthetic compounds that inhibit the activity of PDEs, such as MXTs, which are natural PDE-inhibitors and are currently used as therapy for several non-neoplastic diseases, with adequate pharmacokinetic, pharmacodynamic, and security profiles [11]. Due to their pharmacological properties, MXTs could become an adjuvant therapy against GBM [12,13]. However, their potential effectiveness against GBM remains controversial (Table 1). In the following paragraphs, we will discuss the current status of natural MXTs as therapy for GBM in vitro and in vivo (Figure 1).

## 2. Phosphodiesterases in Glioblastoma (GBM)

PDEs are enzymes that hydrolyze the phosphodiester bond in the cyclic Guanidine Monophosphate (cGMP) and cyclic Adenosine Monophosphate (cAMP), lowering their intracellular concentration. These nucleotides act as second intracellular messengers and participate in cellular metabolism, cell growth, differentiation, and proliferation. They also participate in functions such as reproduction, cardiac function, vision, inflammation, and oncogenesis [24].

The PDE gene family has 11 different members (PDE1 to PDE11) with 21 genes that encode up to 100 different subtypes of proteins. They are grouped by their substrate specificity: cAMP-specific are PDE4, PDE7, and PDE8; cGMP-specific are PDE5, PDE6, and PDE9; the rest use both cAMP and cGMP [12,24].

Alterations in PDE activity contribute to tumorigenesis, reducing the levels of the cyclic nucleotides, which are described in malignant cells [25,26]. In GBM, overexpression of PDE4A1 correlates with reduced levels of cAMP [27,28]. On the other hand, PDE5 expression was associated with prolonged overall survival and the inhibition of PDE5 induced a more aggressive phenotype in vitro [7]. Breast and colon cancer cells treated with PDE inhibitors elevate their intracellular cAMP levels, which induce apoptosis, decreased cell migration, and growth arrest [12].

There is not enough information to define the role of cyclic nucleotides or PDE in GBM (Table 2). Some reports point to cyclic nucleotides as pro-oncogenic signals, and the regulation of PDEs constitutes a therapeutic alternative [12]. However, there are also reports that show that PDEs are related to an overall benefit to the patient. It is important to elucidate the exact role of cyclic nucleotides to propose their inhibitors as candidates for adjuvant therapy in GBM.

## 3. Methylxanthines

MXTs are natural alkaloids and secondary metabolites in various botanical species (*Camellia sinensis* L., *Theobroma cacao*, and *Coffea* sp.) such as caffeine, theophylline, and theobromine. They occur in dietary products like tea, coffee, chocolate, and energy drinks [60,61].

MXTs are clinically used in the treatment of various diseases, such as obesity, hyperlipidemia, chronic obstructive pulmonary disease, asthma, peripheral vascular disease, and apnea of prematurity [62]. Their use is under investigation in the treatment of cancer and neurodegenerative diseases. Recently, their potential role as antineoplastic has gained attention [12,60,63].

MXTs bind to adenosine receptors [64], which participate in several cellular functions, and their expression correlates with tumor survival, chemoresistance, grade, and cancer cell survival [65,66].

Adverse effects (AE) of MTXs are dizziness, irritability, nervousness, tremors, sleep difficulty, diarrhea, nausea, and vomiting [67]. The most relevant AEs are cardiovascular, arrhythmias and cardiac arrest. Caffeine and theophylline show similar AEs, whereas theobromine requires higher doses to become toxic [60]. For caffeine, 15 mg/L is the threshold for toxicity in humans; doses up to 20 g can lead to death secondary to cardiac arrhythmias, ventricular fibrillation, or kidney failure [68]. Administration of MTXs to pregnant women requires special attention [67,69].

### 3.1. Caffeine

Caffeine (1,3,7-trimethylxanthine) is extracted from the *Coffea* sp. and from the leaves of some teas. The physiological effects of caffeine include central nervous system (CNS) stimulation, smooth muscle relaxation, and tachycardia [70]. Caffeine has a wide absorption via the digestive system, easy passage of the blood-brain barrier (BBB), and good systemic distribution. Once absorbed, 95% of caffeine is metabolized to paraxanthine (85%), theobromine (10%), and theophylline (5%) [71,72].

Caffeine is used for treatment of apnea of prematurity in infants between 28 and 33 weeks of gestational age; it is effective in other pathologies such as fatigue [73]; headache; migraine [72,74]; orthostatic hypotension [75]; and other neurological diseases such as depression [76], brain injury [77], anxiety [78], and Alzheimer’s [79] and Parkinson’s disease [80].

Another promising clinical application of caffeine is in neuro-oncology. The population with the greatest caffeine consumption showed a reduced prevalence of gliomas [81]. Caffeine has been tested as a therapeutic adjuvant in the treatment of gliomas; Stewart et al. showed the beneficial effect of the combination of caffeine plus cytosine arabinoside and cisplatin [19]. Caffeine potentiates cisplatin-induced and camptothecin-induced apoptosis (G2 phase shortening) in glioma cell lines [20], increasing the subG1 stage [21].

Sinn et al. [22] showed that caffeine can sensitize radiation-resistant cells (U87MG, T98G, and U373MG), due to the activation of the checkpoint in the G1 phase. This effect may be explained by the capability of caffeine to inhibit phosphoinositide 3-kinase (PI3K), down-regulating the PI3K/Akt pathway and inducing apoptosis.

Caffeine can induce apoptosis and cell cycle arrest via different mechanisms; apoptosis can be induced by inhibition of PI3K, by activation of caspase-3, poly(ADP-ribose) polymerase (PARP), or forkhead box protein O1 (FoxO1) [23,82,83]. Caffeine in combination with tetrandrine (a natural alkaloid isolated from the root of the plant Radix Stephania Tetrandrae) induces apoptosis, independent of caspase activation [84]. Additionally, epigenetic modifications such as decreased activity of histone deacetylase 1 (HDAC1) and increased activity of histone acetyltransferase have been related to induction apoptosis by caffeine [85]. The MXT arrest the cell cycle in G0/G1 suppressing Rb phosphorylation [82], whereas caffeine also induces shortening of the G2 phase [20].

An important aspect of the malignancy in GBM is its ability to infiltrate healthy brain parenchyma. It has been proposed that caffeine can induce a cytotoxic effect, preventing the invasive behavior of glioma cells. These effects may result of interactions of caffeine with intracellular calcium. They can be due to the inhibition of the inositol triphosphate receptor type 3 (IP3R3), which has been associated with longer survival in a mouse xenograft model of GBM [86].

Focal adhesion complexes are used by GBM cells to invade and migrate. This complex is regulated by Rho-associated protein kinase (ROCK); caffeine inhibits migration of glioma cells, apparently through phosphorylation of p21, glycogen synthetase kinase 3 beta, and the ROCK pathway [23]. This decreased migration correlates with an experimental reduction of tumor growth [21,87].

Another important feature of GBM is angiogenesis. Hypoxia induces the expression of hypoxia-inducible factor 1 alpha (HIF-1a) protein, which promotes the expression of vascular endothelial growth factor (VEGF), inducing the formation of new blood vessels. Caffeine inhibits the expression of HIF-1α and VEGF [88].

In summary, caffeine can block several pathological pathways in GBM. Caffeine induces apoptosis, blocks the cell cycle, prevents migration and invasion of neoplastic cells, and inhibits angiogenesis.

### 3.2. Theophylline

Theophylline (1,3-dimethylxanthine) is extracted mainly from the tea plant (*Camellia sinensis* L.) and yerba mate (*Ilex Paraguariensis*) [89,90]. Theophylline stimulates the CNS and induces bronchodilatation [91,92]. Theophylline decreases metastasis, inflammation, and therapy-resistance in cancer cells. The role of theophylline has been related to the inhibition of PI3K, an activated pathway in cancer that favors metastasis and resistance to treatment [93]. In addition, it acts as an immunomodulator due to the activation of the HDAC-2 protein, which suppresses the expression of inflammatory genes [63,91,94]. This drug reduces survival and proliferation of A-172 and U87MG glioma cells [14]. Theophylline has been tested in other types of cancer, such as lung cancer. Domvri et al. showed the synergic effect between PDE inhibitors and chemotherapeutic agents (docetaxel, cisplatin, and carboplatin) [95]. In cervical and breast cancer cells, theophylline mediates alternative splicing through suppression of serine/arginine-rich splicing factor 3 (SRSF3) and its target genes, these effects alter the status of p53 isoforms, which contributes to cancer progression in these cells [96].

### 3.3. Theobromine

Theobromine (3,7-dimethylxanthine) is derived from *Theobroma cacao*, which is a metabolite from caffeine and is found in chocolate, tea, and some species of *Camellia sinensis* L. [60]. It has been shown that cocoa and its metabolites are antioxidant and cardiovascular protectors; theobromine might also possess anti-tumor and anti-inflammatory effects mediated by the elevation of intracellular cAMP levels due to the non-selective inhibition of PDE4A1 [18].

Theobromine passes the BBB and placental barriers, inducing neurophysiological effects [97] such as inhibition of adenosine receptors in the CNS [90]. However, as it lacks a methyl group, theobromine has a modest metabolic activity, being limited in its distribution in the CNS [60].

In GBM, Sugimoto et al. reported that theobromine inhibits proliferation and promotes cell death in the U87MG cell line, through inhibition of AKT/mTOR and PDE4. It was also shown that cell death induced by theobromine was related to the switch between the activity of ERK, JNK, and p38, inducing apoptosis; theobromine may prevent tumor progression by inhibiting NF-kB-phosphorylation [18].

## 4. Methylxanthines and Cellular Differentiation

The effect of theophylline as a modulator of differentiation was evaluated in GBM by Nagai et al. They reported that theophylline induces morphological changes in human glioblastoma and in malignant glioma cells induced by methylcholanthrene in mouse C57, to a less malignant phenotype [15]. Sato et al. also reported that theophylline induces glial-like morphological changes and expression of a protein marker of glial cells (S-100 protein) after five days of exposure in a mouse glioma cell line. From the sixth day of exposure, viability of this cell population was decreased [16], which could be interpreted as programmed death of differentiated cells.

Glioma cell treatment with N6,2′-O-dibutyryl cAMP (Bt2AMP) and theophylline caused delayed phosphorylation of the signal transducer and the activator of transcription-3 (STAT3) as well as expression of an astrocyte marker, glial fibrillary acidic protein (GFAP) [17].

There are few publications specifically describing differentiation induced by caffeine. The first clue about a possible role in differentiation of caffeine was mentioned by Kreider et al., in 1973; the authors found that non-toxic doses of caffeine can induce morphological changes of mature cells in melanoma cells [98]. In 1986, an epidemiological study in patients with breast cancer showed that high caffeine consumption correlated with an increased degree of cell differentiation in the tissue [99]. The morphological differentiation of neoplastic cells can be induced by the addition of caffeine, in a time-dependent manner, to sensitize the cancer cells to antineoplastic therapies like that with cisplatin, a widely used drug against various types of cancer, including GBM [100].

As can be speculated, information about the role of MTX as a differentiating drug is promising; nevertheless, research about this is limited.

## 5. Conclusions

The design of novel therapies for the treatment of GBM is urgently needed. However, the creation of novel therapeutic substances implies a large investment of time and money. In various fields of pharmacotherapy, there is an alternative—namely, drug repurposing. This is a process in which drugs that are clinically used are explored for other clinical indications. MXTs have beneficial effects in different GBM cell types and in animal models. However, there is limited knowledge about the specificity of these drugs to inhibit PDEs, such as the potential therapeutic use of MTXs against malignant properties of GBM.

MXTs regulate various cell processes such as proliferation, migration, cell death, and differentiation, all of them related to cancer progression, which are potential therapeutic targets in GBM (Figure 1). More research should be conducted on the role of different PDEs in GBM, as well as on the specificity of MXTs for different PDEs, which can lead to defining potential therapeutic applications of MXTs against GBM.

MXTs are classical pharmacological substances, with defined effectiveness and safety profiles, so the repurpose of these drugs is a fair possibility. All MXTs have similar toxicity profiles; side effects are related to the dose, so in order to prevent them, it is important to define the target mechanisms and the different dosages to achieve them.

There are several gaps in our knowledge of the pathological or physiological effects related to specific PDEs and the potential benefits of different MXTs.

## Figures and Tables

**Table 1:** Effect of methylxanthines in glioma.

Main Author (Reference)	Methylxanthine	Type of Study	Relevant Methodology	Relevant Results
Moon et al., 2012 [14]	Theophylline	*In vitro*	A-172 and U87MG cell lines	Reduces the survival and proliferation
Nagai et al., 1971 [15]	Theophylline	*In vitro*	Human glioblastoma cells and glioma cells induced by MC in C57 black mouse	Induces morphological changes
Sato et al., 1975 [16]	Theophylline	*In vitro*	Mouse glioma cell line	Induces glial-like morphological changes and expression of S-100 protein.
Takanaga et al., 2004 [17]	Theophylline	*In vitro*	C6 cell line	N6,2′-O-dibutyryl cAMP (Bt2AMP) and theophylline caused delayed phosphorylation of STAT3 and expression of GFAP.
Sugimoto et al., 2014 [18]	Theobromine	*In vitro*	U87MG cell line	Anti-tumoral and anti-inflammatory effects. Inhibits proliferation and induces apoptosis.
Stewart et al., 1987 [19]	Caffeine	Clinical	25 patients with gliomas Caffeine added to cytosine arabinoside plus cisplatin	Presence of caffeine-induced seizures 48% of the patients responded.
Janss et al., 1998 [20]	Caffeine	*In vitro*	U251 glioma cells	Caffeine reduced the ID50 and ID90 of cisplatin promoting apoptosis.
Chen et al., 2014 [21]	Caffeine	*In vitro*	C6 and U87MG cell lines	Caffeine decreases migration by inhibition of ROCK-focal adhesion complex pathway.
Sinn et al., 2010 [22]	Caffeine	*In vitro*	U87MG, T98G and U373MG cells lines	Inhibits PI3K, downregulating the PI3K/Akt pathway and induces apoptosis.
Ku et al., 2011 [23]	Caffeine	*In vivo*	Mouse xenograft model of GBM	Inhibits of the IP3R3.

ID50, inhibitory dose 50. ID90, inhibitory dose 90. STAT3, signal transducer and activator of transcription 3. MC, methylcholantrene. ROCK, rho-associated protein kinase. PI3K, phosphoinositide 3-kinase. Akt, protein kinase B. IP3R3, inositol triphosphate receptor type 3. RR, relative risk. CI, confidence interval. mL, milliliters. NIH-AARP, National Institutes of Health-American Association of Retired Persons.

**Table 2:** Role of phosphodiesterases on glioblastoma.

Phosphodiesterase	Gene Chromosome	Substrate	Main Function	Participation in GBM	Ref.
PDE1	*PDE1A*, *B*, *C* 2q32.1, 12q13.2, 7q14.3	cAMP and cGMP	Promotes cell proliferation and migration	*PDE1C* is overexpressed on GBM	[29,30,31,32]
PDE2	*PDE2A* 11q13.4	cAMP and cGMP	Regulates endothelial permeability and proliferation and nNOS expression.	*PDE2A* is overexpressed in low grade glioma	[33,34]
PDE3	*PDE3A*, *B* 12q12.2, 11p15.2	cAMP and cGMP	Smooth muscle contraction, insulin signaling, blood vessel formation, and antiapoptotic and anti-inflammatory pathways	N. D.	[35,36,37]
PDE4	*PDE4A*, *B*, *C*, *D* 19p13.2, 1p31.3, 19p13.11, 5p11.2-q12.1	cAMP	Promotes blood vessel formation, monocyte and macrophage activation, and antiapoptotic and anti-inflammatory pathways	PDE4 promotes the tumor growth Hypermethylation of the *PDE4C* promoter is associated with high malignant grade and reduced overall survival	[36,38,39,40,41]
PDE5	*PDE5A* 4q26	cGMP	Regulates cell signaling	PDE5 is overexpression correlates with longer overall survival, and its inhibition induces an invasive phenotype of GBM	[7,42]
PDE6	*PDE6A*, *B*, *C* 5q32, 4p16.3, 10q24	cGMP	Participates in rod and cone photoreceptor function	N. D.	[43,44,45]
PDE7	*PDE7A*, *B* 8q13, 6q23-24	cAMP	Modulation of T-cell proliferation	*PDE7B* overexpression induces tumor growth	[46,47,48,49]
PDE8	*PDE8A*, *B* 15q25.3, 15q13.3	cAMP	Controls T cells and breast cancer cells motility	*PDE8A* expression correlates with an increased overall survival	[50,51,52,53]
PDE9	*PDE9A* 21q22.3	cGMP	Participates in synaptic plasticity and cognitive function	N. D.	[54,55]
PDE10	*PDE10A* 6q26	cAMP and cGMP	Regulates intracellular signaling and controls striatal gene expression	*PDE10A* is deleted on GBM tissue	[56,57,58]
PDE11	*PDE11A* 2q31.2	cAMP and cGMP	Contributes to sperm development	N. D.	[59]

GBM, glioblastoma; Ref., reference; PDE, phosphodiesterase; cAMP, cyclic Adenosine Monophosphate; cGMP, cyclic Guanosine Monophosphate; nNOS, neural Nitric Oxide Synthetase; N. D., no data. *Cursive* for gene names.
